# Biceps Femoris Activation during Hamstring Strength Exercises: A Systematic Review

**DOI:** 10.3390/ijerph18168733

**Published:** 2021-08-18

**Authors:** Luis Llurda-Almuzara, Noé Labata-Lezaun, Carlos López-de-Celis, Ramón Aiguadé-Aiguadé, Sergi Romaní-Sánchez, Jacobo Rodríguez-Sanz, César Fernández-de-las-Peñas, Albert Pérez-Bellmunt

**Affiliations:** 1 Faculty of Medicine and Health Sciences, Universitat Internacional de Catalunya, 08017 Sant Cugat del Vallès, Spain; lllurda@uic.es (L.L.-A.); nlabata@uic.es (N.L.-L.); carlesldc@uic.es (C.L.-d.-C.); sergiromani@uic.es (S.R.-S.); jrodriguezs@uic.es (J.R.-S.); 2 ACTIUM Functional Anatomy Group, Universitat Internacional de Catalunya, 08195 Sant Cugat del Vallès, Spain; 3 Institut Universitari per a la Recerca a I’Atenció Primària de Salut Jordi Gol i Gurina (IDIAPJGol), 08007 Barcelona, Spain; 4 Department of Nursing and Physical Therapy, Universitat de Lleida, 25003 Lleida, Spain; 5 Department of Physical Therapy, Occupational Therapy, Rehabilitation and Physical Medicine, Universidad Rey Juan Carlos, 28933 Madrid, Spain; cesar.fernandez@urjc.es

**Keywords:** hamstring, muscle injury, biceps femoris, muscle activity, electromyography

## Abstract

Background: The aim of the study was to systematically evaluate the biceps femoris long head activation across cross-sectional hamstring strength exercise studies. Methods: A systematic review design was followed. The search strategy conducted in PubMed, Cochrane Library, and Web of Sciences databases found a total of 3643 studies. Once inclusion and exclusion criteria were applied, 29 studies were finally included in this systematic review. A total of 507 participants and 114 different exercises were analyzed. Exercises were evaluated individually and grouped into several categories: Nordics, isokinetic exercises, lunges, squats, deadlifts, good mornings, hip thrusts, bridges, leg curls, swings, hip and back extensions, and others. Results: Results showed the isokinetic and Nordic exercises as the categories with highest biceps femoris activation (>60% of Maximal Voluntary Isometric Contraction). Nordic hamstring exercise ankle dorsiflexion was the exercise that achieved the highest biceps femoris long head activation (128.1% of its Maximal Voluntary Isometric Contraction). Conclusions: The results from this systematic review suggest that isokinetic and Nordic exercises seem to be the best option to activate biceps femoris long head. Future studies evaluating the implementation of these exercises in prevention programs are needed.

## 1. Introduction

Hamstring strain injury (HSI) is one of the most common injuries in sports involving high-intensity sprinting, acceleration, and decelerations [1]. Injury rates of the hamstring muscles ranges from 6% to 29% of all injuries in track and field, soccer, Australian football, rugby, basketball, or cricket [2].

The biceps femoris long head (BFlh) is the most affected muscle, involving around 80% of all HSIs [3]. Moreover, around 30% of HSIs are recurrent injuries [4]. In fact, previous HSI is a primary risk factor of a re-injury across literature [5,6]. Research into BFlh injuries continues to develop and has led to a better understanding of the problem [7].

Hamstring strength is one of the muscle properties that has received more attention in current research both as preventive and performance-enhancing strategy [8,9,10]. It seems that hamstring strength deficit is a good predictor of HSI [6]. In addition, strength has been also found to be a risk factor for preventing HSI in recent prospective cohort studies [8,11,12]. At the same time, hamstring strength seems to be positively correlated with athletic performance [13,14]. In fact, mostly all sport teams include hamstring strength exercises as part of their performance-enhancing and prevention strategy [15,16,17,18]. Improving athletic performance is a common objective in the sports field. The hamstring muscles are essential in many aspects of sports practice [18]. Hamstring muscle activity is higher than any other muscle group during a sport maneuver as essential to sport as sprinting [18]. For this reason, hamstring training exercises are a crucial component of sports performance.

Interestingly, there are a lot of strength exercises described and used in the current literature, making it difficult to determine the most appropriate when developing specific programs for the training of these muscles.

Several studies had compared BFlh muscle activation by using the surface electromyography (sEMG) activity level between hamstring strength exercises [19,20,21]. sEMG has been shown to be a good instrument to determine muscle activation levels during strength exercises [22]. sEMG has been used to categorize exercise intensity, and therefore, assisting sport coaches and physiotherapists when selecting the most appropriated exercise [21,23]. Previous studies have systematically reviewed muscle activation during commonly used strength exercises in the gluteus maximus (GMax) [24,25], which is another important hip extensor. However, no systematic review has previously investigated the differences in biceps femoris muscle activation across strength exercises. Therefore, the purpose of the current systematically review was to evaluate biceps femoris sEMG during the most common strengthening exercises in a healthy population.

## 2. Materials and Methods

### 2.1. Registration

The systematic review was performed in accordance with the Preferred Reporting Items for Systematic Reviews and Meta-Analyses (PRISMA) statement checklist [26]. The systematic review protocol was registered in the PROSPERO database with ID: CRD42020183079.

### 2.2. Information Sources and Search

The search strategy was developed following the PICO (Population, Intervention, Comparison, Outcomes) strategy.

Population: healthy adults without lower extremity injury;

Intervention: biceps femoris activation during hamstring strength exercises;

Comparison: no comparison due to specific cross-sectional study designs;

Outcome: biceps femoris muscle activity as assessed with sEMG was primary outcome. The “last 10 years” filter was used in all three databases. Keywords used to develop the search strategy are shown in Table 1.

Databases used in the current systematic review were PubMed, Cochrane Library, and Web of Science. Furthermore, the lists of references from the studies included were checked to find other studies meeting inclusion criteria. The final search was performed on 1 May 2020. A complete PubMed database search strategy example is shown in Table 2.

### 2.3. Eligibility Criteria and Study Selection

The inclusion criteria for studies included in this systematic review were as follows: (1) cross-sectional design; (2) healthy individuals; (3) evaluation of hamstring strength exercises; (4) providing data about sEMG of hamstring muscles; (5) providing data normalized by MVIC; (6) specific data about BFlh or “lateral hamstring musculature”; (7) English or Spanish language; and (8) published during the last 10 years. Studies were excluded if they included: (1) elderly people; (2) did not provide percentage activation data; or (3) did not specify the normalization method. Moreover, if studies provided data via bar charts, the corresponding author of the article was contacted, and the means and standard deviations of each exercise were requested.

Titles and abstracts were screened by two independent authors (SRS and LLL). In case of discrepancy, a third author (APB) was consulted. The Cohen’s Kappa index was used in order to assess the inter-rater agreement. Landis et al. [27] categorized the Kappa Statistic as <0.00 as poor inter-rater agreement, 0.00–0.20 as slight, 0.21–0.40 as fair, 0.41–0.60 as moderate, 0.61–0.80 as substantial, and 0.81–1.00 as almost perfect inter-rater agreement.

### 2.4. Data Collection Process

The following data were extracted for studies included in this systematic review: (1) Author’s last name and year of publication; (2) sample size; (3) exercises performed; (4) normalization method; (5) electrode placement; and (6) testing load.

### 2.5. Outcomes

As primary outcome, the average sEMG root mean square (RMS) expressed as a percentage of MVIC for BFlh /lateral hamstring musculature was chosen.

This review categorized hamstring exercises according to muscle activation of the hamstring muscles following Macadam and Feser [28]: low activation (0 to 20% Maximal Voluntary Isometric Contraction, MVIC), moderate activation (21 to 40% MVIC), high activation (41 to 60% MVIC), or very high activation (61% or greater MVIC).

### 2.6. Risk of Bias of Individual Studies

The National Institutes of Health Quality Assessment Tool for Observational Cohort and Cross-sectional Studies was used to assess the methodology quality of the included studies [29]. This tool contains 14 quality questions assessing internal validity (population, sample size, statistical analysis, and outcome measures). Questions must be answered as “Yes”, “No”, “cannot determine (CD)”, “not applicable (NA)”, or “not reported (NR)”. Cross-sectional studies automatically scored “not applicable” on criteria 6, 7 and 10 (Supplementary Table S1). One point was obtained only if the question was answered as “Yes”. All questions were equally weighted in overall quality assessment results.

## 3. Results

### 3.1. Study Selection

**Figure 1:**
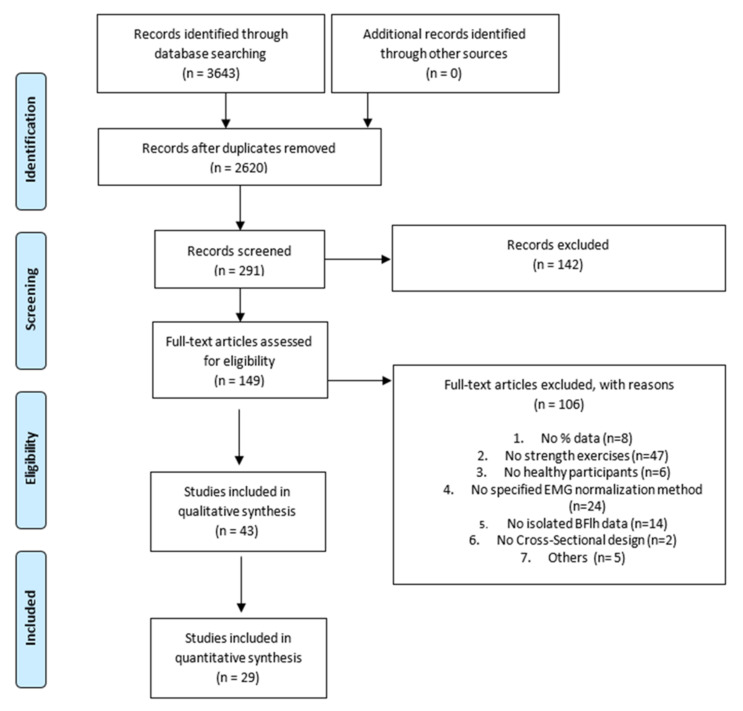
Preferred Reporting Items for Systematic Reviews and Meta Analyses (PRISMA) flow diagram.

The search strategy found a total of 3643 studies (PubMed: 987; ScienceDirect: 2083; Cochrane Library: 573). Two thousand six hundred and twenty studies were initially included after checking for duplicates. After title screening, 291 studies were considered for full abstract screening. One hundred and forty-two studies were excluded after reading the abstract, so 143 full-text articles were assessed for eligibility. Full-text screening was carried out, and 43 studies finally met the inclusion criteria. The reasons for the exclusion of 106 studies were as follows: did not provide data as %MVIC (*n* = 8), did not assess hamstring strength exercises (*n* = 47), did not include healthy participants (*n* = 6), did not specify sEMG normalization method (*n* = 24), did not provide isolated BFlh/lateral hamstring data (*n* = 14), no cross-sectional design (*n* = 2), others (*n* = 5). Finally, 43 studies were included in the qualitative analysis. From these 43, twenty did not provide numerical data other than bar charts. Thus, the corresponding authors from these papers were contacted. Twenty-nine studies were finally included in the quantitative analysis. The Cohen’s Kappa index showed an “almost perfect” agreement (k = 0.87). The detailed study selection and reasons for excluded articles can be found in the PRISMA flow chart (Figure 1).

### 3.2. Study Characteristics

Studies involved a total of 507 participants with an age ranging from 19 to 30 years. The exercises mostly commonly assessed in the studies were swings, dead lifts, Nordic hamstring exercises (NHE), squats, leg curls, lunges, good mornings, hip thrusts, and hip extensions. The majority of the studies normalized hamstring EMG data by using MVC in prone position with the knee flexed at 45° and the sEMG electrodes placed following the Surface Electromyography for the Non-Invasive Assessment of Muscles (SENIAM) guideline [30].

The testing load differed among studies, but bodyweight was the more commonly used load. Table 3 summarizes the characteristics of the included studies.

### 3.3. Quality Assessment 

The studies included in this systematic review showed a mean score of 5.75 from a total of 11 points (from 4 to 8 points) in the National Institutes of Health Quality Assessment Tool for Observational Cohort and Cross-sectional Studies [29].

### 3.4. Muscle Activation

**Figure 2:**
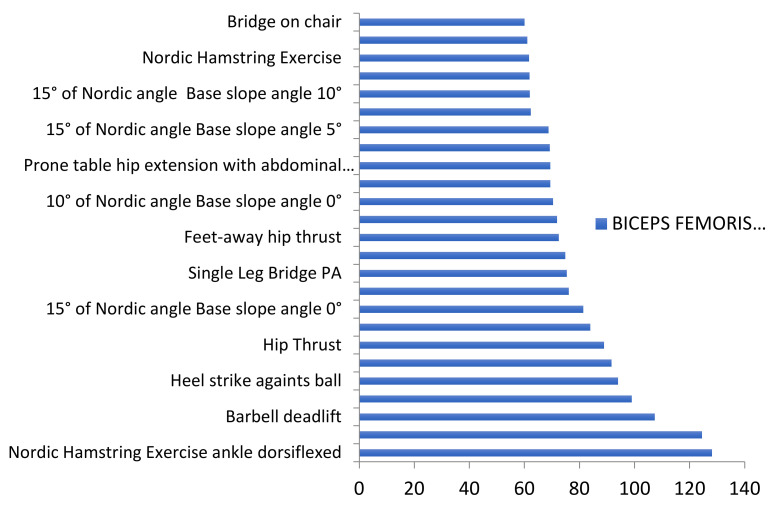
Exercises with very high BF activation levels.

A total of 114 exercises were assessed across articles included in this review. Two different evaluations were performed across exercises. Firstly, we assessed them and ordered them from lower to higher biceps femoris activation (based on %MVIC). Secondly, as many studies performed similar exercises, we grouped them into the following categories: Nordics, deadlifts, hip thrust, swing, squats, good mornings, bridges, hip extensions, isokinetic exercises, and lunges (Figure 2). Data regarding the 114 exercises are available in Supplementary Table S2.

This figure highlights both NHEs from Comfort et al. [52] and barbell deadlifts from Andersen et al. [59] with biceps femoris activation higher than 100% MVIC. Moreover, the slip leg exercise and the heel strike against ball exercise from Arias-Poblete et al. [39] achieved 99% and 94% of BFlh activation, respectively.

**Figure 3:**
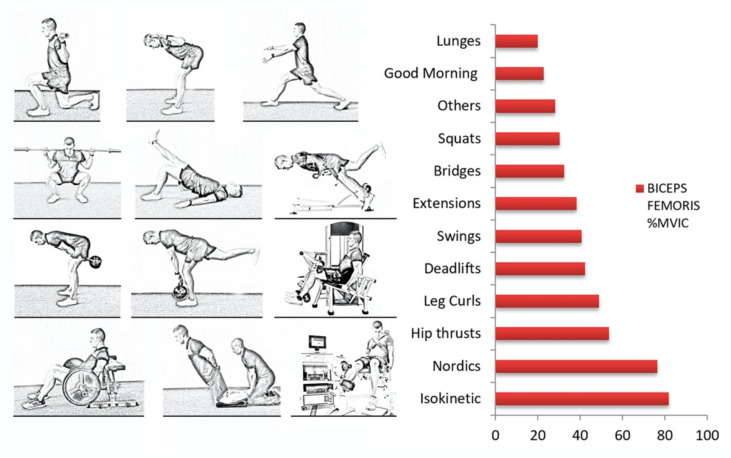
Categories ordered by BF mean activation levels.

The assessment of categories instead of exercises gives more global information about the exercise typology. Moreover, it minimizes a possible bias in one study which could notably alter the results. Figure 3 shows the results of BFlh activation levels by categories.

Very high activation (>60%MVIC).

Isokinetic exercises represented the highest biceps femoris activation with a mean of 81.7%MVIC. However, only two exercises were able to be included in this category. Furthermore, NHE also achieved a very high mean activation of 76.5%MVIC. In this second case, eleven NHEs were evaluated. All of them achieved a mean activation higher than 60%MVIC. In fact, the NHE with ankle dorsiflexion achieved the higher BFlh activation in this systematic review with 128%MVIC.

High activation (from 41% to 60%MVIC).

Three exercise categories showed a mean activation between 41% and 60% of the MVIC: hip thrust (53.50%), leg curl (48.78%), and deadlifts (42.17%).

Moderate activation (from 21% to 40%MVIC).

Swing exercises (40.52%), hip or back extensions (38.19%), glute bridges (32.34%), squats (30.24%), other exercises (28.09%), and good mornings (22.79%) were the six categories that produced a moderate activation of the BFlh muscle.

Low activation (<20%MVIC).

Only the lunge exercise category achieved a mean activation lower than 20%. This category showed a mean BFlh activation level of 19.82% of MVIC.

## 4. Discussion

This systematic review aimed to evaluate the biceps femoris activation among different strength hamstring exercises. The results have shown that the Nordic hamstring exercise with ankle dorsiflexion is the exercise achieving the highest BFlh muscle activation. Moreover, the Nordic hamstring category (all variations) is the second-best exercise for BG activation category. These results may assist coaches, practitioners, or physiotherapists in selecting exercises based on intensity purposes.

The Nordic hamstring exercise has been widely used in the literature. Its first use in research was 2004 when Mjølsnes et al. [60] evaluated its effectiveness in hamstring eccentric strength. The use of NHE to improve hamstring eccentric strength is undoubtable. Several publications [61,62,63] and a recent systematic review and meta-analysis [64] support this idea. Furthermore, the meta-analyses from van Dyk et al. [65] and Al Attar et al. [66] concluded that including the NHE as an injury prevention exercise reduces the rate of HSI. Although some authors have questioned its use [67], current and previous data support the NHE as being associated with the highest levels of muscle BFlh activation.

The isokinetic exercise category was the highest in BFlh activation of all the categories. Isokinetic exercises have been commonly used for testing, for predicting injury risk, and as criteria for return to play after both hamstring and anterior cruciate ligament injury [6,68,69]. However, results from this study also support its use as a potential form of hamstring strengthening due to its very high activation of the biceps femoris muscle.

The results from the two exercises included in the “others” category also need attention. The “slip leg” and “heel strike against ball” exercises are quite different from deadlifts, swings, thrusts, curls, etc. However, they have shown activation levels of 94% and 99% of MVIC, placing them at the top fourth and fifth position in the ranking. Thus, these results would recommend these exercises as part of high-intensity hamstring training.

On the other hand, lunges were the lowest BFlh activation category. These results are due to the fact that lunge exercises are commonly used for improving gluteus muscle but not hamstring strength. In fact, a systematic review evaluating gluteus maximus activation during common strength exercises found lunges variations as high activity (>60% of MVIC) exercises [24]. Thus, this kind of exercise should not be performed when hamstring activation and strength is the objective of the program.

This review focuses on muscle activation during different exercises commonly used to improve sport performance. However, other parameters such as strength, timing, or position may play an important role when choosing exercises [6,70,71]. Malliaropoulus et al. [6] supported the idea that hamstring strength exercises should also be oriented to specific sport demands. The highest activation of the biceps femoris occurs during the late swing phase and early stance phase of high speed running [71]. Thus, both open and closed kinetic chain exercises should be supported to reproduce the high demands of sprinting during sport maneuvers. Furthermore, hamstrings present a dual role in both hip and knee joints at the same time while running [72]. For this reason, both hip and knee dominant exercises should be supported to “simulate” their function during sprinting [6,73].

Although muscle activation gains are usually understood to be a prediction of strength gains, this relationship has not been scientifically proven [70]. This difference between muscle activation and strength must be taken into account when interpreting current results. Nevertheless, it should be also considered how this muscle is integrated in relation to other synergistic muscles involved in the particular or specific movement pattern [70].

### 4.1. Limitations

We should recognize some limitations from this systematic review. First, some studies evaluating the impact of exercise selection on muscle activity had to be excluded because they did not normalize their sEMG data by MVIC test. Second, in this systematic review, we did not consider exercise loads due to the high heterogeneity and the fact that it could alter the results. The high variability in the normalization methods makes difficult to compare directly several studies. Finally, current data are based only on healthy people; we do not currently know if muscle activation of injured sports players would exhibit a similar tendency. Furthermore, this review did not specify whether the participants were professional athletes or amateurs, nor did it specify the type of sport practiced. Therefore, it cannot be assured that these results can be extrapolated to a specific sport or to a specific level of sport expertise.

### 4.2. Sports and Clinical Applications

The strength training BFlh programs must be effective and specific to the necessity of sports athletes. The effective BFlh training programs are mandatory to enhance athletic performance. Exercise selection plays an important role to achieve this. This systematic review provides evidence regarding which exercises activate more the biceps femoris, which is one of the main muscles in sports. The results have shown that Nordic hamstring exercises and isokinetic exercises were more efficient to activate the BFlh more.

## 5. Conclusions

The results from this systematic review allow coaches, athletic trainers, and physical therapists to classify different exercises from low to high muscle activation of the BFlh. These data could implement a progressive training strategy to improve hamstrings strength and therefore athletic performance. However, although this review involves data from more than five hundred participants, individual anatomy variations could modify muscle activations during exercises. Thus, an anamnesis and physical examination of each participant is always recommended in order to individualize exercise selection. 

## Figures and Tables

**Table 1:** Keywords used for the search strategy.

Exercise	Muscle Activity	Muscle
Strength	Mucle development	Biceps femoris
Exercise	Myogenesis	Hamstring
Weight bearing	Myofibrillogenesis	Semitendinosus
Force	Hypertrophy	Semimembranosus
	Electromyography	Posterior thigh
	Excitation	Knee flexor
	Activation	Hip extensor
	EMG	
	Activity	

**Table 2:** Example of search strategy in PubMed database.

((((((hamstring[Title/Abstract] OR biceps femoris[Title/Abstract] OR semitendinosus[Title/Abstract] OR semimembranosus[Title/Abstract] OR “posterior tight”[Title/Abstract] OR “knee flexors”[Title/Abstract] OR “hip extensors”[Title/Abstract]))) AND ((“muscl* development”[Title/Abstract] OR myogenesis[Title/Abstract] OR myofibrillogenesis[Title/Abstract] OR hypertroph*[Title/Abstract] OR electromyogra*[Title/Abstract] OR excitation[Title/Abstract] OR emg[Title/Abstract] OR activity[Title/Abstract] OR activation[Title/Abstract]))) AND ((strengt*[Title/Abstract] OR exercis*[Title/Abstract] OR weight-bearing[Title/Abstract] OR force[Title/Abstract]))))

**Table 3:** Characteristics of included exercises.

Study	Quality Score	Sample Size (Age)	Exercises (Category)	Exercises (Detailed)	Normalization Method	Electrode Placement	Testing Load
Jeon 2016 [31]	6	16 (23.4 ± 2.2)	Hip extensions	Prone hip extension Prone table hip extension Prone table hip extension with knee flexion	According to the guidelines of Kendall et al. [32]	70% on the line extending between the ischial tuberosity and lateral epicondyle	No specified
Del Monte 2020 [33]	6	14 (30 ± 3.9)	Swing	Squat swing Hip hinge swing Double knee extension swing	Prone position with the knee flexed to 90°	According to SENIAM guidelines	Maximum mass the participant could swing for a cadence of 35–40 repetitions/min during the participant’s typical training sessions and ranged from 16 to 48 kg
Lyons 2017 [34]	6	14 (21.5 ± 2.03)	Swing	Swing Snatch swing Clean swing	Prone position with the knee flexed to 70°	Lateral aspect of the thigh 67% of the distance between the trochanter and popliteal fossa, starting at the trochanter	Load for each individual exercise that could be performed for 8–10 repetitions with a good technique. It ranged from 4.5 to 32 kg
Monajati 2017 [35,36]	5	10 (22 ± 4.7)	Nordic Hamstring Exercise Ball leg curl	Nordic hamstring exercise Leg ball curl	Prone position with the knee flexed to 45°	According to SENIAM guidelines	Bodyweight
Lehecka 2017 [36]	4	28 (23.43 ± 2.28)	Bridge	Single-leg bridges different positions	Prone position with the knee flexed to 45°	According to SENIAM guidelines	Bodyweight
Marshall 2010 [37]	5	14 (24.1 ± 1.7)	Others Hip extensions Bridges	Swiss ball rolls Swiss ball hip extension Swiss ball praying mantis Swiss ball single leg squat Prone hold Swiss Ball hold and crunch Swiss ball bridge	No specified	No specified	Bodyweight
Khaiyat 2018 [38]	4	12 (20.10 ± 1.10)	Lunges Others Bridge Squats	Double-leg raise Forward lunge Glute bridge Sit-up Squat	Prone position with the knee flexed to 45°	According to SENIAM guidelines	Bodyweight
Arias-Poblete 2019 [39]	6	30 (21.8 ± 1.46)	Deadlifts Swings Nordic Hamstring Exercises Bridges Others	Single-leg deadlift Swing Nordic hamstring exercise Bridge on chair Prone bridge Bridge in lateral position Strike Neutral back bridge Slip leg Heel strike against ball Four supports with extended arms and legs Scissors held in lateral position Single bridge	No specified	According to SENIAM guidelines	Bodyweight
Collazo 2020 [40]	5	7 (29.4 ± 4.6)	Hip thrusts	Hip thrust Pull hip thrust Rotation hip thrust Feet-away hip thrust	Prone position with the knee flexed to 45°	According to SENIAM guidelines	40% 1 RM
Contreras 2016 [41]	4	13 (28.9 ± 5.1)	Hip thrusts	Barbell hip thrust Band hip thrust American hip thrust	Prone position with the knee flexed to 45°	According to SENIAM guidelines	10 RM
Severini 2018 [42]	5	11 (22.2 ± 1.38)	Deadlifts Others Hip extension	Extender Diver Glider	Knee flexion	According to SENIAM guidelines	Bodyweight
Mausehund 2018 [43]	5	13 (24.9 ± 2.9)	Squats	Rear foot elevated split squat Single-leg squat Split squat	Prone position with the knee flexed to 45°	According to SENIAM guidelines	6–8 RM
Hegyi 2019 [19]	6	19 (26.1 ± 3.2)	Deadlifts Hip extensions Good morning Leg curl Bridges	Straight-knee bridge Upright hip extension conic-pulley Slide leg curl Prone leg curl 45° hip extension Bent-knee bridge Cable pendulum Unilateral Romanian deadlift Good morning	Lay prone with the trunk and hip fixed to the dynamometer bench in neutral position	Midpoint along the ischial tuberosity—popliteal fossa distance	12 RM
Vigotsky 2015 [44]	6	15 (24.6 ± 5.3)	Good morning	Good morning	Prone position with the knee flexed to 45°	On the muscle bellies, parallel with muscle fibers	Submaximal 1 RM
Lawrence 2019 [45]	7	20 (26.8 ± 7.8)	Back extension	Back extension Reverse hyperextension	While the subject was in the top position of hip extension	According to SENIAM guidelines	No specified
Kim 2013 [46]	7	22 (23.5 ± 4.92)	Hip extension	Floor hip extension Round foam roll hip extension	No specified	2 cm from the lateral border of the thigh and two-thirds of the distance between the trochanter and the back of the knee	No specified
Jeon 2017 [47]	8	16 (25.4 ± 4.2)	Hip extension	Prone table hip extension Prone table hip extension with abdominal drawing-in Prone table hip extension with the abdominal drawing-in maneuver with the flexed contralateral knee joint on a chair	According to the guidelines of Kendall et al. [33]	Two-thirds of the distance along the line extending between the ischial tuberosity and lateral epicondyle	Bodyweight
Kawama 2020 [48]	6	14 (19.6 ± 1.0)	Deadlift	Adduction double-leg deadlift Neutral double-leg deadlift Abduction double-leg deadlift 20° internal rotation double-leg deadlift 20° External rotation double-leg deadlift 40° External rotation double-leg deadlift	Knee flexion	Over 40 and 60% of the thigh length (the distance between the greater trochanter (0%) and the popliteal crease (100%)) for BFlh	60% of their body mass
Ryu 2012 [49]	6	14 (23.3 ± 3.74)	Bridge	Bridge on stable base Bridge on unstable base	No specified	According to SENIAM guidelines	Bodyweight
Choi 2016 [50]	6	27 (27.8 ± 5.8)	Bridge	Bridge Single bridge Single bridge with hip abduction Single bridge with sling Single bridge with sling and hip abduction	No specified	On the thigh between the knee and buttocks	Bodyweight
Lee 2019 [51]	5	26 (23.15 ± 2.68)	Hip extension	Prone hip extension Prone hip extension with hip abduction and knee flexion	According to the guidelines of Kendall et al. [33]	2 cm from the lateral border of the thigh and two-thirds of the distance between the trochanter and the back of the knee	Bodyweight
Comfort 2017 [52]	6	15 (22.6 ± 2.1)	Nordic hamstring exercise	Nordic hamstring ankle dorsiflexed Nordic hamstring ankle plantar flexed	Prone position with the knee flexed to 45°	Placed at the midline of the muscle belly of both the BF	Bodyweight maximal effort
Park 2019 [53]	7	21 (NR)	Nordic hamstring exercise	10° Nordic hamstring base slope angle 0° 10° Nordic hamstring base slope angle 10° 10° Nordic hamstring base slope angle 15° 15° Nordic hamstring base slope angle 0° 15° Nordic hamstring base slope angle 10° 15° Nordic hamstring base slope angle 15°	No specified	Two-thirds of the distance between the trochanter and the back of the knee	Bodyweight maximal effort
Muyor 2020 [54]	6	20 (24 ± 5.55)	Squat Lunge	Monopodal squat Forward lunge Lateral step-up	Prone position with the knee flexed to 45°	According to SENIAM guidelines	60% of 5 RM
Jónasson 2016 [55]	7	40 (24.1 ± 2.6)	Isokinetic	Isometric knee flexion medial rotation Isometric knee flexion lateral rotation	Prone position with the knee flexed to 45°	According to SENIAM guidelines	5 s isometric
Park 2014 [56]	6	20 (21.94 ± 2.24)	Back extension	Back extension, knee extended, hands on sternum Back extension, knee extended, hands behind head Back extension, knee flexed, hands on sternum Back extension, knee flexed, hands behind head	Prone position with the knee flexed to 45°	At the muscle in the center of the back of the thigh, approximately half the distance from the gluteal fold to the back of the leg	Bodyweight
Contreras 2016 [57]	6	13 (28.9 ± 5.1)	Squats	Front squat Full squat Parallell squat	Prone position with the knee flexed to 45°	According to SENIAM guidelines	10 RM
Narouei 2018 [58]	6	10 (26.1 ± 5.46)	Nordic hamstring exercise	Nordic hamstring exercise	No specified	According to SENIAM guidelines	Maximal effort
Andersen 2018 [59]	5	13 (21.9 ± 1.6)	Hip thrusts Deadlift	Hip thrust Deadlift Hex bar deadlift	Prone position with the knee flexed to 45°	According to SENIAM guidelines	1 RM

## Supplementary Materials

The following are available online at https://www.mdpi.com/article/10.3390/ijerph18168733/s1, Table S1: Risk of Bias of Individual Studies and Table S2: Muscle Activation.
