# Supplementary material for: Biceps Femoris Activation during Hamstring Strength Exercises: A Systematic Review
Source: Int J Environ Res Public Health. 2021 Aug 18;18(16):8733. doi: 10.3390/ijerph18168733 (PMC8393607; doi:10.3390/ijerph18168733)
Supplement: Supplementary file 1 [file ijerph-18-08733-s001.zip › ijerph-1311041-supplementary.pdf]

International Journal of
Environmental Research and Public Health

Table S1. Risk of Bias of Individual Studies.

	Item 1	Item 2	Item 3	Item 4	Item 5	Item 6	Item 7	Item 8	Item 9	Item 10	Item 11	Item 12	Item 13	Item 14	Total Yes
Jeon 2016	yes	yes	NR	yes	yes	NA	NA	NA	yes	NA	yes	NA	NA	NR	6
Del Monte 2017	yes	yes	NR	yes	no	NA	NA	Yes	yes	NA	yes	NA	NA	NR	6
Lyons 2017	yes	yes	NR	yes	no	NA	NA	Yes	yes	NA	yes	NA	NA	NR	6
Monajati 2017	yes	yes	NR	yes	no	NA	NA	NR	yes	NA	yes	NA	NA	NR	5
Lehecka 2017	yes	yes	NR	NR	no	NA	NA	NR	yes	NA	yes	NA	NA	NR	4
Schoenfeld 2015	yes	yes	NR	yes	no	NA	NA	NR	yes	NA	yes	NA	NA	NR	5
Marshall 2010	yes	yes	NR	CD	yes	NA	NA	NR	yes	NA	yes	NA	NA	NR	5
Khaiyesat 2018	yes	yes	NR	cd	no	NA	NA	No	yes	NA	yes	NA	NA	NR	4
Arias 2019	yes	yes	NR	yes	yes	NA	NA	No	yes	NA	yes	NA	NA	NR	6
Collazo 2018	yes	yes	NR	yes	no	NA	NA	No	yes	NA	yes	NA	NA	NR	5
Contreras 2016	yes	yes	NR	NR	no	NA	NA	No	yes	NA	yes	NA	NA	NR	4
Severini 2018	yes	yes	NR	yes	no	NA	NA	No	yes	NA	yes	NA	NA	NR	5
Mausehund 2018	yes	yes	NR	yes	no	NA	NA	No	yes	NA	yes	NA	NA	NR	5
Hegyi 2019	yes	yes	NR	yes	no	NA	NA	Yes	yes	NA	yes	NA	NA	NR	6
Lawrence 2019	yes	yes	yes	yes	no	NA	NA	Yes	yes	NA	yes	NA	NA	no	7
Kim 2013	yes	yes	yes	yes	no	NA	NA	Yes	yes	NA	yes	NA	NA	no	7
Jeon 2016	yes	yes	yes	yes	yes	NA	NA	Yes	yes	NA	yes	NA	NA	NR	8
Kawama 2020	yes	yes	NR	yes	no	NA	NA	Yes	yes	NA	yes	na	NA	NR	6
Ryu 2012	yes	yes	NR	yes	no	NA	NA	Yes	yes	NA	yes	NA	NA	NR	6
Lee 2019	yes	yes	NR	NR	no	NA	NA	Yes	yes	NA	yes	NA	NA	NR	5
Comfort 2017	yes	yes	NR	yes	no	NA	NA	Yes	yes	NA	yes	NA	NA	NR	6
Park 2019	yes	yes	NR	yes	yes	NA	NA	Yes	yes	NA	yes	NA	NA	NR	7
Muyor 2020	yes	yes	NR	yes	no	NA	NA	Yes	yes	NA	yes	NA	NA	NR	6
Jonasson 2016	yes	yes	NR	yes	yes	NA	NA	Yes	yes	NA	yes	NA	NA	NR	7
Park 2014	yes	yes	NR	yes	no	NA	NA	Yes	yes	NA	yes	NA	NA	NR	6
Contreras 2016	yes	yes	NR	yes	no	NA	NA	Yes	yes	NA	yes	NA	NA	NR	6
Narouei 2018	yes	yes	NR	yes	no	NA	NA	Yes	yes	NA	yes	NA	NA	NR	6
Choi 2016	yes	yes	NR	yes	no	NA	NA	Yes	yes	NA	yes	NA	NA	no	6
Vigotsky 2015	yes	yes	NR	yes	no	NA	NA	Yes	yes	NA	yes	NA	NA	no	6
Total															57,586,207

Table S2. Muscle Activation.

REERENCE	EXERCISES	MEAN BF	SD BF
**Comfort 2017**	Nordic hamstring exercise ankle dorsiflexed	128.1	5
**Comfort 2017**	Nordic hamstring exercise ankle plantar flexed	124.5	6.2
**Andersen 2017**	Barbell deadlift	107.3	18.4
**Arias-Poblete 2019**	Slip leg	99	
**Arias-Poblete 2019**	Heel strike againts ball	94	
**Jonasson 2016**	Isokinetic knee flexion lateral rotation	91.57	24.06
**Andersen 2017**	Hip thrust	88.9	18.2
**Andersen 2017**	Hex bar deadlift	83.9	18.2
**Park 2019**	15° of Nordic angle base slope angle 0°	81.32	23.57
**Mausehund 2019**	Rear foot elevated split squat	76.09	37.1
**Lehecka 2017**	Single-leg bridge PA	75.34	24.25
**Monajati 2017**	Nordic hamstring exercise	74.8	20
**Collazo 2018**	Feet-away hip thrust	72.43	33.21
**Jonasson 2016**	Isokinetic knee flexion medial rotation	71.84	40.41
**Park 2019**	10° of Nordic angle; base slope angle 0°	70.33	23.62
**Jeon 2016**	PTHE	69.37	30.01
**Jeon 2016[a]**	Prone table hip extension with abdominal drawing-in	69.37	30.01

**Lehecka 2017**	Single-leg bridge PC	69.18	18.49
**Park 2019**	15° of Nordic angle; base slope angle 5°	68.72	13.23
**Mausehund 2019**	Split squat	62.27	28.4
**Park 2019**	15° of Nordic angle; base slope angle 10°	61.93	18.7
**Park 2019**	10° of Nordic angle; base slope angle 10°	61.82	20.62
**Narouei 2018**	Nordic hamstring exercise	61.68	48.29
**Collazo 2018**	Pull hip thrust	61.07	18.37
**Arias-Poblete 2019**	Bridge on chair	60	
**Mausehund 2019**	Single-leg squat	59.73	28.01
**Lehecka 2017**	Single-leg bridge PD	58.71	19.72
**Lee 2019**	Prone hip extension	55.85	12.58
**Lyons 2017**	Swing	55.7	46.4
**Arias-Poblete 2019**	Nordic hamstring exercise	55	
**Jeon 2016[a]**	Prone table hip extension	54.66	25.8
**Hegyi 2019**	Straight-knee bridge	53.84	11.57
**Marshall 2010**	Swiss ball rolls	53.6	27.9
**Hegyi 2019**	Slide leg curl	53.37	12.33
**Hegyi 2019**	Upright hip extension conic-pulley	52.97	11.01
**Park 2019**	10° of Nordic angle; base slope angle 15°	52.32	14.22
**Arias-Poblete 2019**	Four supports with extended arms and legs	52	
**Jeon 2016[a]**	Prone table hip extension with the abdominal Drawing-in maneuver with the flexed Contraleteral knee join on a chair	50.32	26.19
**Monajati 2017**	Ball leg curl	50.3	25.7
**Lyons 2017**	Snatch swing	45.2	25.9
**Lyons 2017**	Clean swing	45.2	21.7
**Lawrence 2019**	Reverse hyperextension	44.3	19.9
**Contreras 2016**	American hip thrust	44.2	20
**Collazo 2018**	Rotation hip thrust	42.8	19.83
**Hegyi 2019**	Prone leg curl	42.66	10.03
**Collazo 2018**	Hip thrust	41.02	29.89
**Contreras 2016**	Barbell hip thrust	40.8	22.1
**Arias-Poblete 2019**	Single-leg bridge	40	
**Kim 2013**	Floor hip extension	39.12	18.6
**Kawama 2020**	40° external rotation double leg deadlift	37.36	15.79
**Lawrence 2019**	Back hip extension	36.9	25.4
**Contreras 2016**	Band hip thrust	36.8	18
**Hegyi 2019**	Bent-knee bridge	35.75	12.57
**Kim 2013**	Round foam hip extension	35.01	17.43
**Hegyi 2019**	45° hip extension	34.55	9.8
**Severini 2018**	Glider	34.48	23.55
**Kawama 2020**	20° external rotation double-leg deadlift	34.23	10.01
**Kawama 2020**	Abduction double-leg deadlift	32.58	14.78
**Arias-Poblete 2019**	Swing	32	
**Jeon 2016**	Prone hip extension	31.02	15.18
**Severini 2018**	Diver	30.82	17.02
**Park 2014**	Back extension, knee extended, hands behind head	30.5	16
**Vigotsky 2014**	Good morning 90% RM	30.4	(28.0–32.9)
**Park 2014**	Back extension, knee extended, hands on sternum	30.2	14.54
**Kawama 2020**	20° internal rotation double-leg deadlift	29.92	14.08
**Kawama 2020**	Aduction double-leg deadlift	29.91	13.51
**Muyor 2020**	Monopodal squat	29.72	15.22
**Arias-Poblete 2019**	Single-leg deadlift	29	
**Kawama 2020**	Neutral double-leg deadlift	27.95	14.17
**Choi 2016**	Single-leg bridge	27.7	14.7
**Park 2014**	Back extension, knee flexed, hands on sternum	27.26	19.1

**Park 2014**	Back extension, knee flexed, hands behind head	27.12	20.79
**Ryu 2012**	Bridge on unstable surface	26.41	11.71
**Vigotsky 2014**	Good morning 80% RPM	26.4	(23.9–28.8)
**Choi 2016**	Single-leg bridge with hip abduction	26.2	11.9
**Arias-Poblete 2019**	Strike	26	
**Jeon 2016**	PTHEK	25.22	11.4
**Del Monte 2017**	Hip hinge swing	24.5	9.19
**Vigotsky 2014**	Good morning 70% RM	24.4	(21.8–26.6)
**Lehecka 2017**	Single-leg bridge PB	23.49	9.3
**Khaiyat 2018**	Glute bridge	22.4	4.3
**Hegyi 2019**	Cable pendulum	22.25	8.72
**Arias-Poblete 2019**	Neutral back bridge	22	
**Del Monte 2017**	Squat swing	21.69	9.18
**Hegyi 2019**	Unilateral Romanian deadlift	20.88	9.09
**Lehecka 2017**	Single-leg bridge PE	20.84	12.81
**Marshall 2010**	Swiss ball hip extension	20.6	12.9
**Khaiyat 2018**	Lunge	20.5	6.4
**Del Monte 2017**	Double knee extension swing	20.03	11.96
**Vigotsky 2014**	Good morning 50% RM	19.5	(17.2–21.9)
**Vigotsky 2014**	Good morning 60% RM	19.3	(17.0–21.7)
**Ryu 2012**	Bridge on stable sufrace	19.25	9.24
**Muyor 2020**	Forward lunge	19.15	11.14
**Khaiyat 2018**	Squat	19	8.1
**Choi 2016**	Single-leg bridge with sling and hip abduction	18.9	8.3
**Muyor 2020**	Lateral step-up	18.12	10.12
**Choi 2016**	Single-leg bridge with sling	17.8	9.8
**Arias-Poblete 2019**	Prone bridge	17	
**Hegyi 2019**	Good morning	16.77	8.13
**Arias-Poblete 2019**	Scissors held in lateral position	15	
**Contreras 2016**	Parallell squat	14.92	6.64
**Contreras 2016**	Full squat	14.39	6.41
**Lee 2019**	Prone hip extension with hip abduction and knee flexion	14.36	8.36
**Choi 2016**	Bridge	14.3	9.1
**Contreras 2016**	Front squat	13.11	4.7
**Severini 2018**	Extender	11.89	15.9
**Marshall 2010**	Swiss praying mantis	10.6	4.6
**Arias-Poblete 2019**	Bridge in lateral position	10	
**Marshall 2010**	Swiss ball bridge	5.6	5.7
**Khaiyat 2018**	Double leg raise	5.3	1.3
**Khaiyat 2018**	Sit up	5	1.2
**Marshall 2010**	Swiss ball single leg squat	3.6	5.5
**Marshall 2010**	Prone hold	2.2	1
**Marshall 2010**	Swiss ball hold and crunch	2.1	0.8

BF: Biceps femoris; SD: Standard deviation.
